# A prognostic nomogram for distant metastasis in thyroid cancer patients without lymph node metastasis

**DOI:** 10.3389/fendo.2025.1523785

**Published:** 2025-02-17

**Authors:** Xiaoqing Yu, Qin Deng, Xin Gao, Lingyun He, Daixing Hu, Lu Yang

**Affiliations:** ^1^ Department of Breast and Thyroid Surgery, Second Affiliated Hospital of Chongqing Medical University, Chongqing, China; ^2^ Hepatopancreatobiliary Center, The Second Affiliated Hospital of Nanjing Medical University, Nanjing, Jiangsu, China; ^3^ Scientific Research and Education Section, Chongqing Health Center for Women and Children, Chongqing, China

**Keywords:** thyroid cancer, distant metastasis, risk factor, nomogram, overall survival

## Abstract

**Background:**

Despite having negative lymph node (N0) status, thyroid cancer (TC) patients can still experience distant metastasis (DM), which significantly impacts their survival. This study aimed to investigate the prognostic factors for DM in TC patients (N0) and develop a predictive nomogram model for analyzing the prognosis of TC N0 patients with DM.

**Methods:**

Data collected from the Surveillance, Epidemiology, and End Results (SEER) database for 18,504 TC patients (N0) between 2004 and 2015 were analyzed. Univariate and multivariate analyses were used to identify independent prognostic factors for DM in TC N0. These independent factors were used to build a nomogram model to predict overall survival (OS) at 1, 3, and 5 years for TC patients (N0) with DM.

**Results and conclusion:**

This study examined the clinicopathological features associated with the risk and prognosis of DM in TC patients (N0), and successfully established and validated a nomogram capable of predicting OS in individual patients with DM. The nomogram is highly useful for the timely identification of TC patients (N0) at high risk of DM by physicians, enabling individualized survival evaluations and treatment for TC patients with DM (N0).

## 1 Introduction

Thyroid cancer (TC) is the most common malignant endocrine tumor, accounting for approximately 94.5% of all cases (1). Its prevalence has increased rapidly in recent years, with an annual growth rate of around 4%. TC is one of the few malignant tumors whose incidence rate is still rising (2–4). While most TC patients experience minimal distant metastasis (DM) and favorable survival outcomes, a subset of patients without lymph node metastasis still face the challenges of DM and poor prognosis. This subgroup requires further attention and investigation.

DM in TC patients is associated with worse prognoses. A recent study indicates that a small number of TC patients present with DM at diagnosis, a significant cause of TC-related mortality (5). Generally, TC patients primarily experience lymph node metastasis before progressing to DM. Consequently, those without lymph node metastasis are generally classified as low risk. However, a small portion of patients with DM also exhibit negative lymph node status, indicating a higher malignancy grade. Numerous studies have explored the risk factors for DM in TC and assessed overall survival (OS). While DM is commonly considered a late-stage event in cancer progression, evidence suggests that metastasis can occur at early stages in certain tumors or as an advanced event, without requiring differentiation at the primary tumor site (6). This phenomenon, known as metastasis dormancy, is supported by experimental models referred to as ‘tumor dormancy’ (7–9) and ‘cellular dormancy’ (10, 11). Therefore, when a tumor particularly TC, deviates from the traditional metastasis model (enlargement of the primary lesion, infiltration of regional lymph nodes, DM), it generally indicates increased invasiveness and a poorer prognosis (12).

Overall, TC is associated with a favorable prognosis. Clinicians often prioritize local lymph node metastasis over DM before and after diagnosis, particularly in differentiated TC, which significantly impacts staging, prognostic assessment, and treatment planning following surgery. Determining the necessity of DM examinations (such as lung CT, bone scanning, etc.) for all patients and identifying those who warrant further evaluation for DM are crucial considerations not only for diagnosing and treating TC patients but also for managing costs effectively.

Nomograms, widely used for prognostic analysis in cancers like TC and breast cancer (13–15), are effective tools for this purpose. Therefore, this study aims to identify risk factors for DM in TC patients without lymph node metastasis and develop a nomogram model for evaluating prognosis in individuals with DM (N0). Additionally, the accuracy and applicability of the nomogram model were validated. By stratifying patients based on prognosis, clinicians can select appropriate examination, treatment methods, and follow-up procedures.

## 2 Materials and methods

### 2.1 Data source

**Figure 1:**
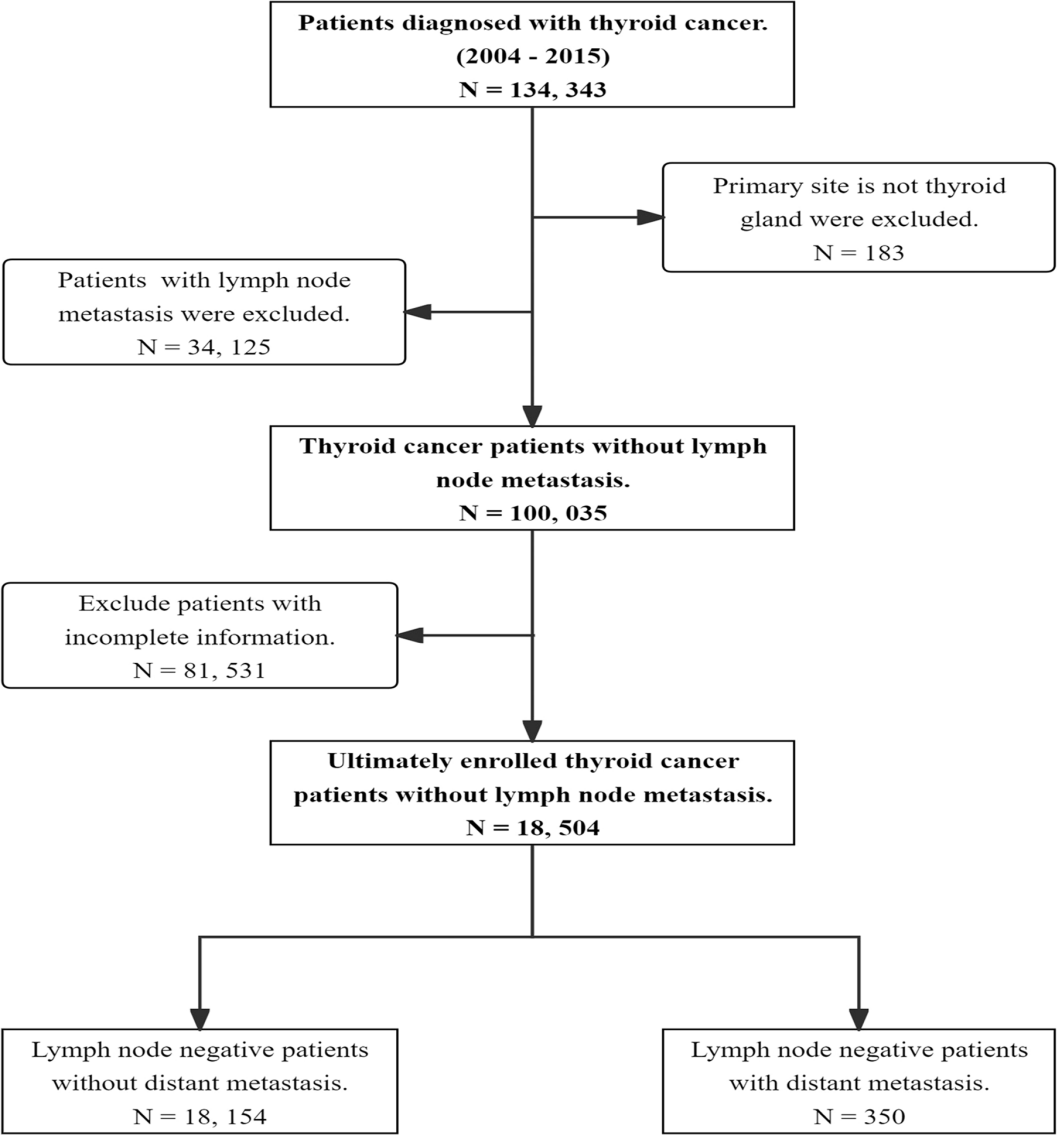
Flow chart illustrating the patient selection process in our study.

The data for this study were obtained from the SEER database (https://seer.cancer.gov/seerstat/) of the National Cancer Institute in the United States (16). The data used in this retrospective study are publicly available. Therefore, the need for informed consent from the patients was waived. No separate ethical approval was required for this study. A retrospective cohort study was conducted using information from the SEER database, which included 134,343 TC patients diagnosed between 2004 and 2015. The inclusion criteria were as follows: (1) Diagnosis of TC between 2004 and 2015; (2) Negative lymph nodes; (3) Pathological types including papillary thyroid cancer (PTC), follicular thyroid cancer (FTC), medullary thyroid cancer (MTC), and anaplastic thyroid cancer (ATC); (4) Active follow- up during the study period. Patients with lymph node metastasis, primary cancer lesions other than thyroid, coexisting tumors, and incomplete clinical and pathological information were excluded. Finally, a total of 18,504 TC patients with N0 status were included in this study. Among these cases, 350 (1.9%) patients (N0) had DM (**Figure 1**).

### 2.2 Statistical analysis

Statistical analysis was performed using SPSS 26.0 (IBM Corp, USA). Univariate and multivariate analyses were conducted to identify independent risk variables for DM or prognostic factors for OS. The Kaplan-Meier (K-M) technique was used to estimate OS, and log-rank tests were used to assess the significance of differences. A p-value of 0.05 (two-sided) indicated statistical significance. Patients from the SEER database were randomly allocated to an internal validation group. The nomogram, calibration curve, and K-M analysis were created using the results of multivariate Cox regression analysis with the R software packages “survival,” “rms,” “sure miner,” and “foreign” (R Foundation, Vienna, Austria, version 3.5.2, http://www.r-project.org). The discrimination performance of the current nomogram was evaluated using Harrell’s C-index.

### 2.3 Ethical approval

SEER data are deidentified before release and do not contain any personally identifying information. As the data are publicly available, no ethical approval is required. We received permission to access the research data file in the SEER program from the National Cancer Institute, USA.

## 3 Results

### 3.1 Clinicopathological characteristics of TC patients without lymph node metastasis status

A total of 18,504 TC patients without lymph node metastasis were included in this study between 2004 and 2015. **Table 1** shows the clinicopathological characteristics of patients with and without DM, along with the distinctions between the two groups. In the TC N0 with DM subgroup, 95 patients (27.1%) were under 55 years old. Among the 350 patients (N0M1), 98 (28.0%) were diagnosed with an ATC pathological type, 91 (26.0%) with an FTC pathological type, 2 (0.6%) with an MTC pathological type, and 159 (45.4%) with a PTC pathological type. The majority of these patients were classified as T4 (143, 40.9%) and grade IV (117, 33.4%). In contrast, within the subgroup without DM, 10,844 individuals (59.7%) were younger than 55 years old, and only 226 patients (1.2%) had an ATC pathological type. The majority of these patients were categorized as T0-1 (11052, 60.9%) and Grade I (14522, 80.0%). Among the N0 patients, 350 cases (1.9%) were identified as coexisting with DM, with 99 cases in the validation cohort.

**Table 1:** Clinicopathological characteristics of the patients.

Variable	Subgroup	Patient demographics (%)	
		M0 (n = 18154)	M1 (n = 350)
**Age**	<55 years	10844 (59.7)	95 (27.1)
	≥55 years	7310 (40.3)	255 (72.9)
**Race**	Black	1511 (8.3)	40 (11.4)
	White	14637 (80.6)	254 (72.5)
	Other	2006 (11.1)	56 (16.1)
**Sex**	Female	14041 (77.3)	235 (67.1)
	Male	4113 (22.7)	115 (32.9)
**Grade**	I	14522 (80.0)	129 (36.9)
	II	2660 (14.7)	41 (11.7)
	III	650 (3.6)	63 (18.0)
	IV	322 (1.7)	117 (33.4)
**Histology**	ATC	226 (1.2)	98 (28.0)
	FTC	1861 (10.3)	91 (26.0)
	MTC	88 (0.5)	2 (0.6)
	PTC	15979 (88.0)	159 (45.4)
**T stage**	T0 - 1	11052 (60.9)	72 (20.5)
	T2 - 3	6497 (35.8)	135 (38.6)
	T4	605 (3.3)	143 (40.9)
**Tumor size**	≤2 cm	12014 (66.2)	99 (28.3)
	2 - 4 cm	3717 (20.5)	74 (21.1)
	≥4 cm	2423 (13.3)	177 (50.6)
**Surgery**	Yes	18033 (99.4)	293 (83.7)
	No	121 (0.6)	57 (16.3)
**Median Income**	<45000$	1791 (9.9)	28 (8.0)
	45000 - 65000$	6995 (38.5)	168 (48.0)
	≥65000$	9368 (51.6)	154 (44.0)

ATC, anaplastic thyroid cancer; FTC, follicular thyroid cancer; MTC, medullary thyroid cancer; PTC, papillary thyroid cancer.

### 3.2 Univariate and multivariate logistic analyses

**Figure 2:**
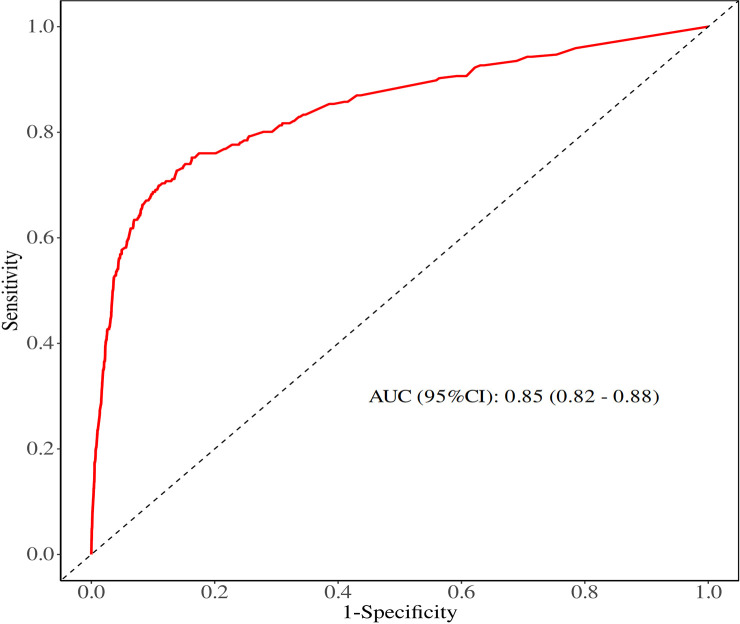
ROC curve of univariate and multivariate logistic analyses evaluating the risk factors for DM.

Logistic analysis was conducted to evaluate the clinical parameters associated with the risk of DM in TC patients with N0 status (**Table 2**). Age at diagnosis (P < 0.001), race (P = 0.001), sex (P = 0.014), grade (P < 0.001), histology (P < 0.001), T stage (P < 0.001), tumor size (P < 0.0001), and surgery (P < 0.001) were found to be significantly associated with DM during the univariate logistic analysis. Subsequently, these eight clinicopathological characteristics were included in the multivariate logistic analysis, which resulted in a satisfactory Receiver operating characteristics (ROC) value of 0.85 (95% confidence interval (CI): 0.82 - 0.88) for predicting the risk of DM in patients with negative lymph node status (**Figure 2**). Specifically, the findings indicated that age ≥55years [Odds Ratio (OR) = 2.11, 95% CI:1.55 - 2.88; <0.001], histology (PTC: OR = 0.47, 95% CI: 0.23 - 0.96, P = 0.037), grade (P < 0.001), T stage (P < 0.001) and surgery (P < 0.001) were independent predictors of DM.

**Table 2:** Univariate and Multivariate Logistic Analyses of Risk Factors of DM.

Variables	Subgroup	Univariable		Multivariable	
		P	OR (95%CI)	P	OR (95%CI)
**Age**	<55 years		1.00 (Reference)		1.00 (Reference)
	≥55 years	**<.001**	4.03 (3.03 ~ 5.34)	**<.001**	2.11 (1.55 ~ 2.88)
**Sex**	Female		1.00 (Reference)		1.00 (Reference)
	Male	**<.001**	1.84 (1.41 ~ 2.40)	0.311	1.16 (0.87 ~ 1.56)
**Race**	Black		1.00 (Reference)		1.00 (Reference)
	White	**0.014**	0.61 (0.41 ~ 0.90)	0.062	0.66 (0.43 ~ 1.02)
	Other	0.552	1.15 (0.72 ~ 1.85)	0.251	1.36 (0.81 ~ 2.28)
**Histology**	ATC		1.00 (Reference)		1.00 (Reference)
	FTC	**<.001**	0.13 (0.09 ~ 0.19)	0.496	1.29 (0.62 ~ 2.68)
	MTC	**0.001**	0.04 (0.01 ~ 0.27)	0.338	0.35 (0.04 ~ 2.99)
	PTC	**<.001**	0.02 (0.02 ~ 0.03)	**0.037**	0.47 (0.23 ~ 0.96)
**Grade**	I		1.00 (Reference)		1.00 (Reference)
	II	0.231	1.32 (0.84 ~ 2.09)	0.673	1.11 (0.69 ~ 1.76)
	III	**<.001**	12.92 (9.08 ~ 18.38)	**<.001**	5.14 (3.42 ~ 7.74)
	IV	**<.001**	36.98 (26.64 ~ 51.34)	**0.005**	3.34 (1.43 ~ 7.82)
**Tumor size**	≤2 cm		1.00 (Reference)		1.00 (Reference)
	2 - 4cm	**<.001**	2.75 (1.91 ~ 3.94)	0.661	0.88 (0.50 ~ 1.55)
	≥4 cm	**<.001**	9.43 (6.97 ~ 12.77)	0.530	1.19 (0.69 ~ 2.06)
**T stage**	T0 - 1		1.00 (Reference)		1.00 (Reference)
	T2 - 3	**<.001**	3.46 (2.45 ~ 4.88)	**0.031**	1.88 (1.06 ~ 3.33)
	T4	**<.001**	36.76 (25.74 ~ 52.51)	**<.001**	4.53 (2.20 ~ 9.36)
**Surgery**	Yes		1.00 (Reference)		1.00 (Reference)
	No	**<.001**	29.53 (19.76 ~ 44.12)	**<.001**	2.45 (1.45 ~ 4.15)
**Median income**	<45000$		1.00 (Reference)		
	45000 - 65000$	0.092	1.51 (0.93 ~ 2.43)		
	≥65000$	0.808	1.06 (0.66 ~ 1.72)		

ATC, anaplastic thyroid cancer; FTC, follicular thyroid cancer; MTC, medullary thyroid cancer; PTC, papillary thyroid cancer; OR, odds ratio; 95% CI, 95% confidence interval. The p-value indicates a significant difference (p < 0.05). The bold p-value indicates a significant difference (p < 0.05).

### 3.3 Univariate and multivariate cox analyses

**Figure 3:**
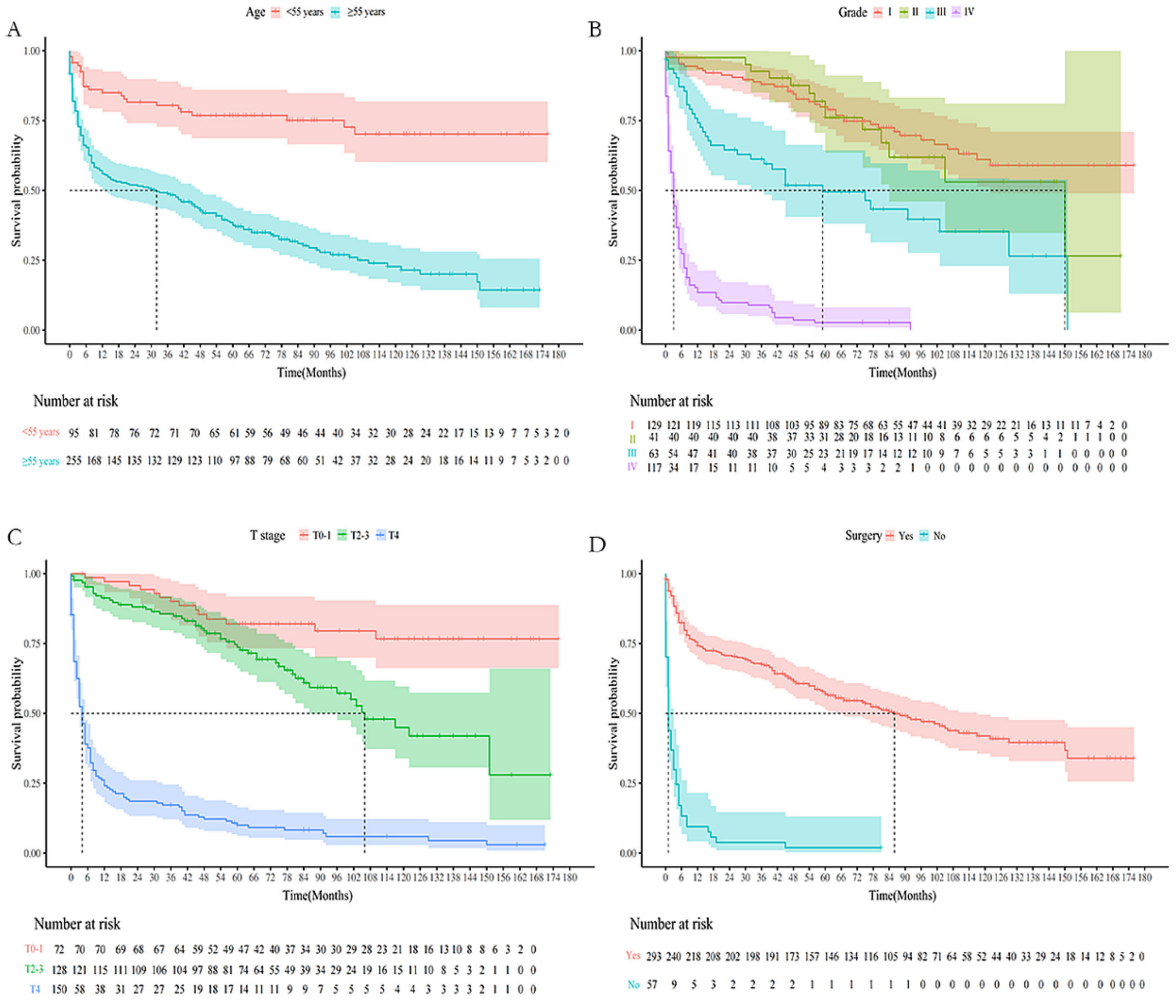
K-M survival curves predicting OS in lymph-node-negative thyroid cancer patients with DM. **(A)** Age; **(B)** Grade; **(C)** T stage; **(D)** Surgery.

The baseline data comparison between the training group and the validation group patients on various indicators showed that this difference was not statistically significant (**Table 3**). Relevant variables from the univariate Cox regression analysis were included in the multivariate Cox regression analysis to determine the independent risk factors for 1-, 3-, and 5-year OS in TC patients (N0) with DM during the follow-up. The multivariate Cox regression analysis identified age at diagnosis (P < 0.001), grade (P < 0.001), T stage (P = 0.014), and surgery (P < 0.001) as significant prognostic variables. Specifically, elderly age (≥55 years: OR = 2.99; 95% CI: 1.86 - 4.80; P < 0.001), grade (III: OR = 1.02, 95% CI: 1.09 - 3.76; IV: OR = 4.63, 95% CI: 1.92 - 11.18), T stage (T4: OR = 2.73, 95% CI:1.23 - 6.06) and surgery (No: OR = 2.53, 95% CI: 1.63 - 3.93, P < 0.001) were identified as independent risk factors for OS in this subgroup (**Table 4**). Furthermore, four factors (P ≤ 0.05) from the multivariate Cox proportional hazard model were used to construct the K-M survival curves to assess the survival probability and cumulative hazard in patients with different variables (**Figure 3**).

**Table 3:** Baseline demographic and clinical characteristics of TC patients (N0) with DM in the training and validation groups.

Variables	Subgroup	Validation cohort (n = 99)	Training cohort (n = 251)	Statistic	P
**Age, n(%)**	<55 years	21 (21.21)	74 (29.48)	χ²=2.46	0.117
	≥55 years	78 (78.79)	177 (70.52)		
**Sex, n(%)**	Female	66 (66.67)	169 (67.33)	χ²=0.01	0.905
	Male	33 (33.33)	82 (32.67)		
**Race, n(%)**	Black	7 (7.07)	33 (13.15)	χ²=2.79	0.247
	White	74 (74.75)	180 (71.71)		
	Other	18 (18.18)	38 (15.14)		
**Histology, n(%)**	ATC	27 (27.27)	71 (28.29)	-	0.920
	FTC	28 (28.28)	63 (25.09)		
	MTC	0 (0.00)	2 (0.80)		
	PTC	44 (44.45)	115 (45.82)		
**Grade, n(%)**	I	43 (43.44)	86 (34.26)	χ²=3.10	0.376
	II	9 (9.09)	32 (12.75)		
	III	15 (15.15)	48 (19.12)		
	IV	32 (32.32)	85 (33.87)		
**Tumor size, n(%)**	≤2 cm	28 (28.29)	71 (28.28)	χ²=0.11	0.948
	2 - 4 cm	22 (22.22)	52 (20.72)		
	≥4 cm	49 (49.49)	128 (51.00)		
**T stage, n(%)**	T0 - 1	18 (18.18)	54 (21.52)	χ²=2.03	0.363
	T2 - 3	44 (44.45)	91 (36.25)		
	T4	37 (37.37)	106 (42.23)		
**Surgery, n(%)**	Yes	79 (79.80)	214 (85.26)	χ²=1.55	0.213
	No	20 (20.20)	37 (14.74)		
**Median income, n(%)**	<45000$	6 (6.06)	22 (8.76)	χ²=1.45	0.485
	45000 - 65000$	52 (52.53)	116 (46.22)		
	≥65000$	41 (41.41)	113 (45.02)		

ATC, anaplastic thyroid cancer; FTC, follicular thyroid cancer; MTC, medullary thyroid cancer; PTC, papillary thyroid cancer; χ², Chi-square test, -: Fisher exact.

**Table 4:** Univariate and Multivariate Cox Analyses of Risk Factors for OS.

Variables	Subgroup	Univariable		Multivariable	
		P	OR (95%CI)	P	OR (95%CI)
**Age**	<55 years		1.00 (Reference)		1.00 (Reference)
	≥55 years	**<.001**	3.70 (2.32 ~ 5.88)	**<.001**	2.99 (1.86 ~ 4.80)
**Sex**	Female		1.00 (Reference)		
	Male	0.052	1.39 (1.00 ~ 1.95)		
**Race**	Black		1.00 (Reference)		
	White	0.884	0.96 (0.60 ~ 1.56)		
	Other	0.595	0.85 (0.45 ~ 1.57)		
**Histology**	ATC		1.00 (Reference)		1.00 (Reference)
	FTC	**0.002**	2.07 (1.32 ~ 3.25)	0.560	1.16 (0.71 ~ 1.90)
	MTC	**0.004**	8.17 (1.95 ~ 34.20)	0.283	2.39 (0.49 ~ 11.70)
	PTC	**<.001**	15.25 (9.67 ~ 24.05)	0.103	1.81 (0.89 ~ 3.68)
**Grade**	I		1.00 (Reference)		1.00 (Reference)
	II	0.341	1.40 (0.70 ~ 2.78)	0.905	1.05 (0.50 ~ 2.17)
	III	**<.001**	3.30 (1.92 ~ 5.67)	**0.027**	2.02 (1.09 ~ 3.76)
	IV	**<.001**	19.54 (11.72 ~ 32.58)	**<.001**	4.63 (1.92 ~ 11.18)
**Tumor size**	≤2 cm		1.00 (Reference)		1.00 (Reference)
	2 - 4 cm	**0.003**	2.23 (1.31 ~ 3.81)	0.664	1.17 (0.57 ~ 2.42)
	≥4 cm	**<.001**	3.63 (2.31 ~ 5.72)	0.184	1.57 (0.81 ~ 3.06)
**T stage**	T0 - 1		1.00 (Reference)		1.00 (Reference)
	T2 - 3	**0.048**	1.91 (1.01 ~ 3.64)	0.468	1.30 (0.64 ~ 2.62)
	T4	**<.001**	11.72 (6.50 ~ 21.15)	**0.014**	2.73 (1.23 ~ 6.06)
**Surgery**	Yes		1.00 (Reference)		1.00 (Reference)
	No	**<.001**	6.94 (4.60 ~ 10.46)	**<.001**	2.53 (1.63 ~ 3.93)
**Median income**	<45000$		1.00 (Reference)		
	45000 - 65000$	0.469	0.81 (0.47 ~ 1.42)		
	≥65000$	0.073	0.60 (0.34 ~ 1.05)		

ATC, anaplastic thyroid cancer; FTC, follicular thyroid cancer; MTC, medullary thyroid cancer; PTC, papillary thyroid cancer; OR, odds ratio; 95% CI, 95% confidence interval. The p-value indicates a significant difference (p < 0.05). The bold p-value indicates a significant difference (p < 0.05).

### 3.4 Construction and validation of the prediction nomogram

**Figure 4:**
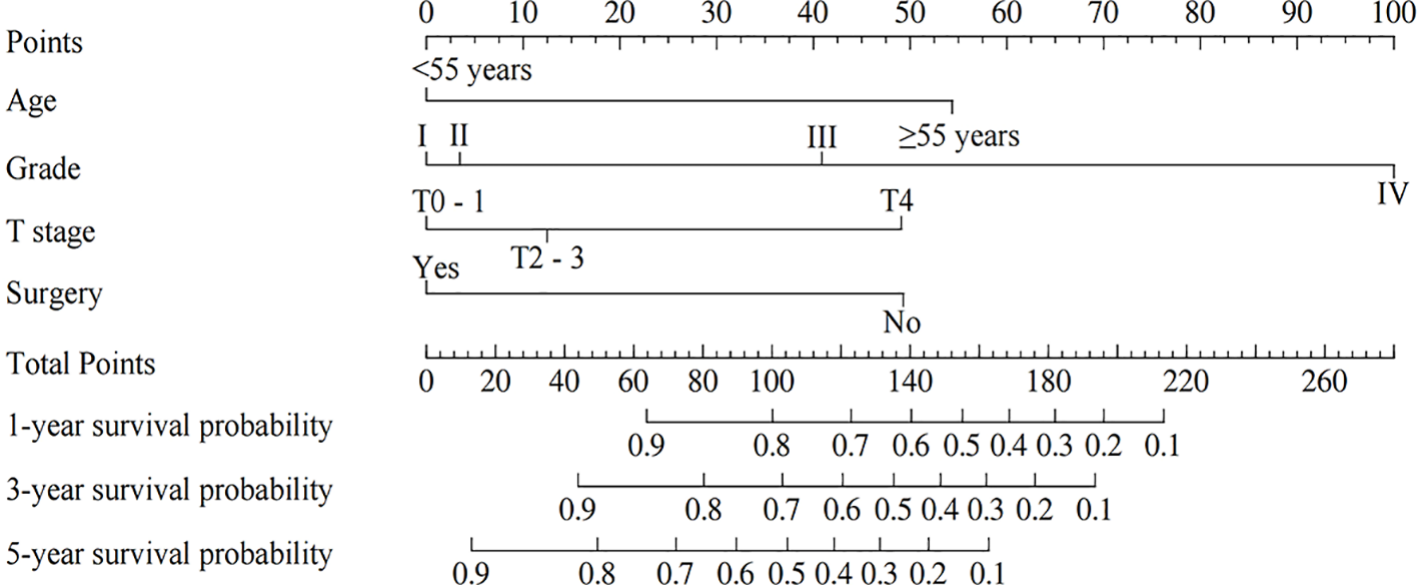
Nomogram predicting the probability of OS.

**Figure 5:**
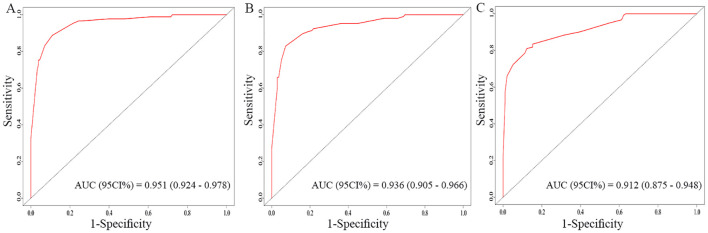
Receiver operating characteristics (ROC) curves of the nomogram for the 1-year, 3-year, and 5-year in the training cohort **(A–C)**.

**Figure 6:**
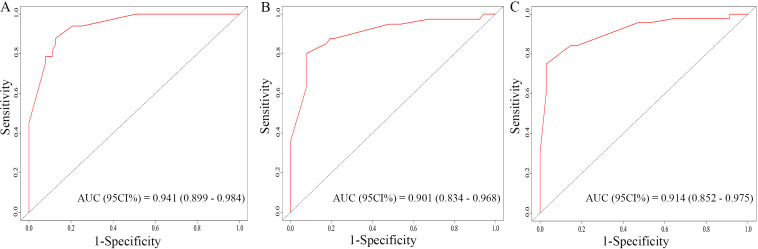
ROC curves of the nomogram for the 1-year, 3-year, and 5-year in the validation cohort **(A–C)**.

**Figure 7:**
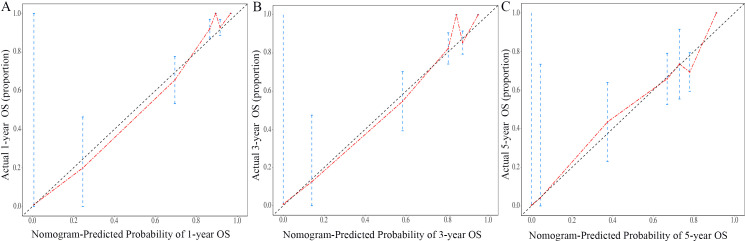
Calibration curves of the nomogram for the 1-year, 3-year, and 5-year in the training cohort **(A–C)**.

**Figure 8:**
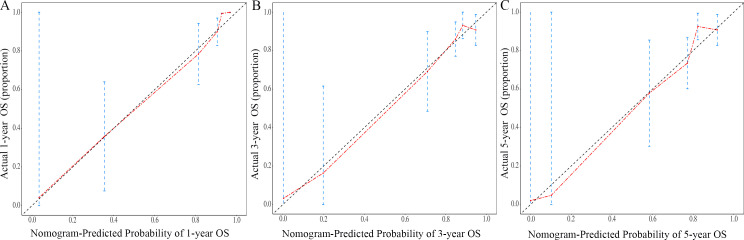
Calibration curves of the nomogram for the 1-year, 3-year, and 5-year in the validation cohort **(A–C)**.

**Figure 9:**
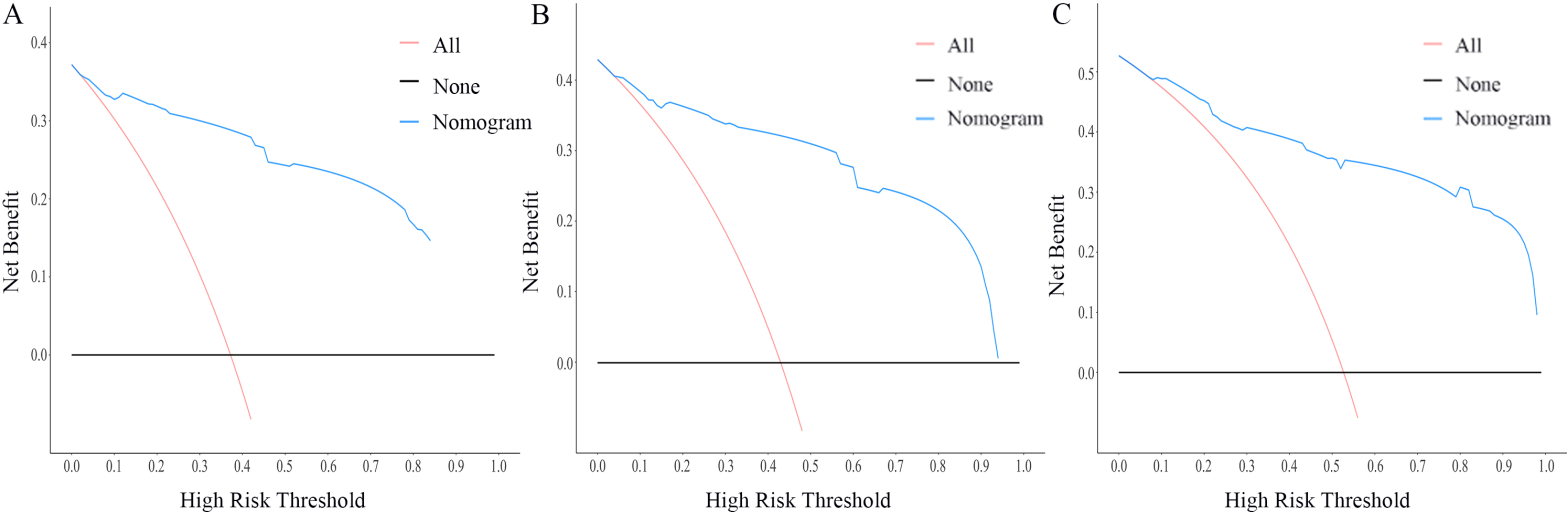
DCA curves of the nomogram for the 1-year, 3-year, and 5-year in the training cohort **(A–C)**.

**Figure 10:**
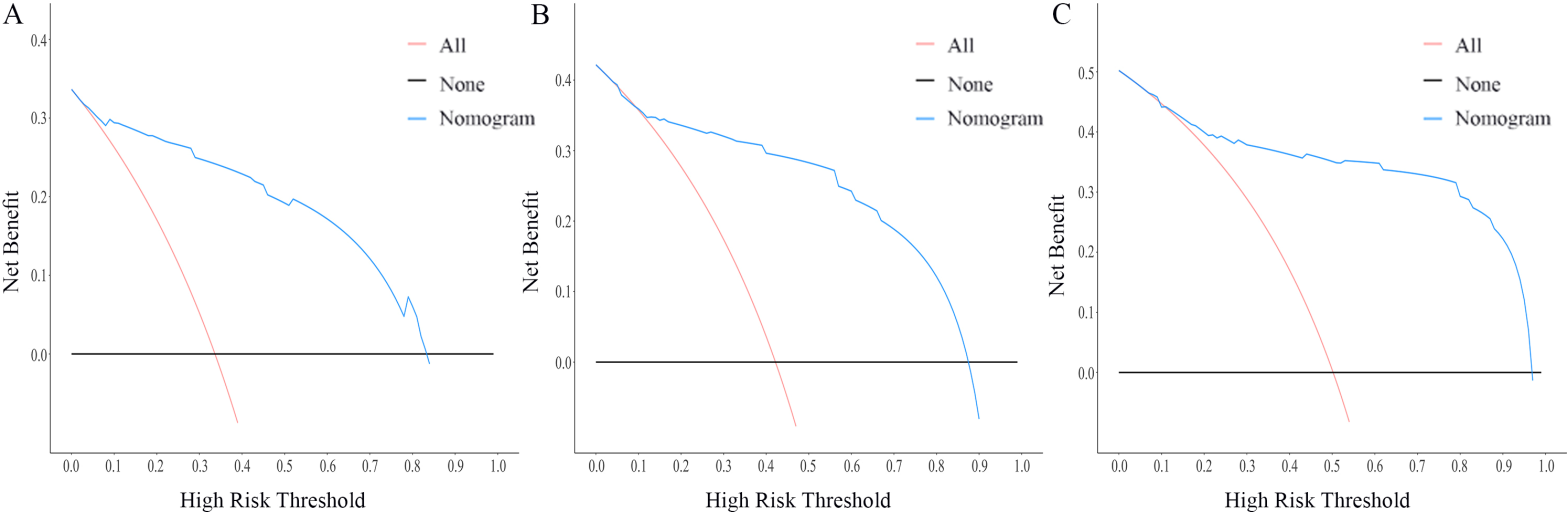
DCA curves of the nomogram for the 1-year, 3-year, and 5-year in the validation cohort **(A–C)**.

The nomogram for OS was constructed based on the independent prognostic variables obtained from the multivariate Cox regression analysis (**Figure 4**). Each variable represented a specific number of points on the scale, and the total score could be computed by summing the points for all variables for an individual patient. In the training cohort, the Area Under Curve (AUC) of the 1-, 3-, and 5-year OS ROC were 0.951, 0.936, and 0.912, respectively, indicating good predictive ability (**Figures 5A–C**). The constructed nomogram was further validated using an internal validation cohort of 162 cases. The validation cohort data also showed significant discrimination, with an AUC of 0.941 for predicting 1-year OS (**Figure 6A**), an AUC of 0.901 for predicting 3-year OS (**Figure 6B**), and an AUC of 0.914 for predicting 5-year OS (**Figure 6C**). To further evaluate the nomogram’s accuracy, the calibration curves for the likelihood of OS revealed a high agreement between the predicted and observed outcomes for the 1-, 3-, and 5-year time points (**Figures 7**, **8**). To assess the clinical applicability of the nomogram, we performed Decision Curve Analysis (DCA) (**Figures 9**, **10**), which measures the net benefits of clinical decisions. The Y-axis represents net profit, while the X-axis represents the threshold for high-risk prediction. The horizontal green solid line represents the net benefit of assuming that all cases are negative, and the diagonal red solid line represents the net benefit of assuming that all cases are positive. The DCA shows that when the threshold probability is greater than 0.1, the model predicts a higher net benefit value, indicating good clinical prediction performance of the model.

## 4 Discussion

TC accounts for 2.3% of all new cancer cases in 2022, according to SEER, with a 5-year relative survival rate of 98.4% (17). Furthermore, TC is expected to surpass colorectal cancer and become the fourth most prevalent cancer by 2030, ranking second only to breast, prostate, and lung cancers (18). While the OS rate of TC patients has improved due to early detection and corresponding treatment (19, 20), the management of DM remains challenging, with a 10-year OS as low as 32%. Therefore, this study aimed to analyze the clinicopathological features of TC patients without lymph node metastases but with DM. The findings revealed associations between age at diagnosis, grade, histology, T stage, surgery, and the presence of DM in patients without lymph node metastasis. These findings can assist clinicians in better understanding the clinical manifestations and risk factors of TC patients with DM and N0, enabling early identification, improved risk stratification, and appropriate treatment to improve OS.

It is widely recognized that the overall prognosis for TC, especially differentiated TC, is generally favorable. Advances in physical examinations and imaging technology have led to increased detection of early-stage TC, particularly those with tumor diameters less than 1cm. These patients often do not exhibit lymph node metastasis, and the preferred surgical approach chosen by clinicians is usually unilateral lobectomy and central lymph node dissection (21, 22). However, our study analyzing the SEER database, which included 18,504 patients, identified 350 cases of M1, accounting for nearly 2% of the study population. While this percentage may not initially appear significant, it is important to consider the rising incidence of TC and the fact that N0 patients with DM often experience more rapid disease progression and have a poorer prognosis. Therefore, the presence of N0M1 patients cannot be overlooked.

Currently, there is very limited research on the clinical treatment and outcomes of TC patients with DM, with most of the literature consisting of individual case reports. Thus, our study aimed to analyze clinicopathological variables of the 2% of patients (N0M1), with the goal of identifying potential risk factors and evaluating the prognosis of DM. By stratifying patients based on the nomogram, clinicians can tailor treatment and follow-up strategies. Specifically, for high-risk patients, preoperative imaging of potential metastatic sites, such as lung CT, bone scanning, etc., may allow early detection and timely intervention, improving prognosis.

Additionally, for high-risk individuals, individualized adjustments to surgical methods and postoperative treatment strategies should be made to optimize patient outcomes. Although N0M1 patients accounted for only 2% of the total cohort in this study, pathological analysis showed that 30.2% (98/324) of ATC patients were N0M1 patients, 4.7% (91/1952) of FTC patients, 2.2% (2/90) of MTC patients, and approximately 1.0% (159/16138) of PTC patients. These findings emphasize the relatively higher proportion of N0M1 patients among ATC cases, highlighting the critical importance of precise treatment for high-risk N0M1 patients across different pathological types. Consensus guidelines typically recommend total thyroidectomy and regional lymph node dissection as the primary treatment for patients with DTC and DM. Surgical intervention is performed for metastatic lesions that are amenable to cure, followed by radioactive iodine (RAI) therapy using I-131 (22). TSH suppression therapy is administered to stable or slowly progressing asymptomatic patients. For those who cannot be cured by surgery or RAI, tyrosine kinase inhibitors (TKIs) such as sorafenib and lenvatinib are used, with evidence showing that TKI therapy extend the survival of patients with distant metastatic DTC (23–25). In the case of MTC, the primary focus lies in treating the primary lesion. For hereditary MTC, total thyroidectomy is typically the initial approach. In sporadic MTC cases, where the lesion often affects both sides, it is commonly recommended to opt for total thyroidectomy as the initial surgical intervention. However, the necessity of surgical treatment for DM in MTC remains controversial. Treatment options for MTC include targeted therapy and radiation. Targeted drugs like vandetanib and cabozantinib have shown efficacy in some cases by slowing tumor growth (26, 27). As for ATC, the 2021 guidelines from the American Thyroid Association emphasize that early evaluation of tumor mutations is crucial for expanding treatment options. The treatment strategy for ATC also encompasses chemotherapy and radiation therapy, with personalized targeted therapy, while personalized targeted therapy utilizing tumor genome information has progressively become the predominant treatment approach (28).

Hence, for TC patients at a high risk of DM, early and precise tumor staging and comprehensive systemic assessment are essential in formulating personalized treatment plans. However, the overall favorable prognosis of TC may sometimes lead surgeons to overlook comprehensive preoperative assessments. As a result, some N0M1 patients may not receive the recommended treatment of complete thyroidectomy followed by RAI, which can negatively impact their prognosis and OS (29). Hence, the findings of this study provide valuable guidance for clinical decision-making. For instance, when encountering patients aged ≥55 years, those with high-grade tumors, MTC, ATC, or advanced T stage in clinical practice, it is crucial to enhance preoperative evaluations to avoid missed diagnoses during the initial treatment. Such improvements can significantly influence the patient’s staging, treatment approach, and ultimate prognosis.

However, it is important to recognize the limitations of this study, which should be addressed in future research. Firstly, this study is retrospective in nature, introducing inherent selection bias. Secondly, the predictor of pathological types had a slightly lower weight on prognosis, which seems to differ from conventional understanding. This discrepancy could be due to significant variations in case numbers among the different pathological types included. If the number of cases is sufficient, subgroup analysis should also be performed for different pathological types. Thirdly, despite the SEER database containing approximately 28% of population-based cancer registries, other potential predictors such as thyroid function, gene status (BRAF, RET, RAS and P53, etc.), the extent of surgery and angioinvasion were not included in the analysis. Studies indicate that genes like BRAF are associated with an increased risk of extrathyroidal extension, lymph node metastasis, advanced disease, and recurrence (30, 31). Furthermore, angioinvasion is also closely linked to DM and may predict the efficacy of systemic RAI therapy (32, 33). Incorporating these factors can significantly enhance the predictive accuracy of the model, enabling clinicians to formulate more precise treatment strategies. Lastly, the lack of an external validation cohort is a notable limitation, limiting the reliability and clinical applicability to some extent. Therefore, future studies should aim to include more cases and predictors, as well as collect data from multiple centers to enhance credibility and applicability.

## 5 Conclusion

In conclusion, the N0 status with DM is a unique scenario that requires intensive study. This study is the first to identify potential clinicopathological features associated with DM in N0 patients and construct a prognosis nomogram for patients diagnosed with DM. Although there are some limitations in this study, the relevant statistical analysis and prediction model have shown good predictive effects, assisting clinicians in estimating the risk of DM and predicting prognosis. It is widely believed that with the inclusion of more data and predictors in future studies, the nomogram will demonstrate even greater clinical applicability.

## Data Availability

Publicly available datasets were analyzed in this study. This data can be found here: https://seer.cancer.gov/seerstat/.
